# Health Coaching for Weight Loss Among Overweight and Obese Individuals in Saudi Arabia: A Retrospective Analysis

**DOI:** 10.7759/cureus.41658

**Published:** 2023-07-10

**Authors:** Rabab A Aldhamin, Ghadeer Al-Ghareeb, Ahmed Al Saif, Zahra Al-Ahmed

**Affiliations:** 1 Preventive Medicine, Al-Ahsa Health Cluster, Al-Ahsa, SAU; 2 Primary Healthcare, Qatif Health Network, Qatif, SAU; 3 Keep Well Unit, Model of Care Department, Eastern Health Cluster, Dammam, SAU

**Keywords:** obesity, saudi arabia, weight loss, lifestyle, coaching

## Abstract

Background

Health coaching is an increasingly used strategy to help in adopting lifestyle changes for weight loss. While Saudi Arabia has one of the highest obesity prevalences worldwide, research on lifestyle interventions for weight loss is limited.

Aim

We aimed to investigate the effectiveness of health coaching for weight loss among the Saudi population in real-world primary healthcare settings.

Methods

This is a retrospective observational study. Secondary data from the health coach national program in the Eastern Health Cluster were retrieved. Obese and overweight individuals aged 15 years or older with weight-related goals who completed at least 12 weeks of coaching were included in the analysis. The primary outcomes are weight change (kg) and weight change percent (%) of the initial weight. We further compared the weight change% between different follow-up methods (i.e., physical, virtual, and hybrid) and studied the factors associated with -5% weight loss.

Results

In total, 465 participants were included in the analysis, with a female predominance (66.2%) and a median initial weight of 90 kg (interquartile range (IQR): 77, 101). The median follow-up duration was 127 days (IQR: 101, 157), and the median total number of coaching sessions was three (IQR: 2, 5). The mean weight change was -2.68 kg (95% confidence interval (CI): -3.12, -2.24), p<0.001. Comparing each follow-up group, no statistically significant difference was found when controlling for number of visits (p=0.059). The adjusted means for weight change% were -3.77%, -2.59%, and -2.54% for hybrid, physical, and virtual visits, respectively. Factors that were associated with achieving at least -5% weight loss were male sex (adjusted odds ratio (aOR)=1.87, 95% CI: 1.16, 3.02), five or more total coaching visits (aOR=5.23, 95% CI: 2.88, 9.50), longer follow-up duration (aOR=1.09, 95% CI: 1.03, 1.15), and having a weight management goal (aOR=4.5, 95% CI: 1.63, 12.45) as the reason for initial coaching visit.

Conclusion

We found statistically significant weight change among clients who completed 12 weeks of coaching in primary care settings. The findings in this paper contribute to the importance of lifestyle interventions for weight loss among the Saudi population. However, stronger controlled studies are needed to confirm this finding.

## Introduction

Background/rationale

Obesity is a well-known risk factor for many chronic diseases, namely type 2 diabetes, hypertension, coronary heart disease, cancers, and other diseases [1]. The World Obesity Federation, in their World Obesity Atlas (2023), estimates that the prevalence of obesity is projected to increase globally from 14% in 2020 to 20% in 2030 [2]. In Saudi Arabia, the estimated prevalence was even higher than that reported worldwide, exceeding 20% in 2019 [3]. This trend is predicted to continue, reaching up to 57% among Saudi adults in 2035 [2]. A number of modalities are used to treat obesity, including lifestyle modification, pharmacological treatment, and surgical interventions [4]. Lifestyle modification is an essential component in obesity management; however, several factors can lead to poor adherence, such as low motivation and negative thoughts [5].

One strategy to help clients in adopting a healthy lifestyle is health coaching [6,7]. The term "health coaching" is defined by Wolever et al. [8] as "a patient-centered approach wherein patients at least partially determine their goals, use self-discovery or active learning processes together with content education to work toward their goals, and self-monitor behaviors to increase accountability, all within the context of an interpersonal relationship with a coach". They further define the health coach as "a healthcare professional trained in behavior change theory, motivational strategies, and communication techniques, which are used to assist patients to develop intrinsic motivation and obtain skills to create sustainable change for improved health and well-being" [8]. Thus, as the definition implies, health coaching mainly focuses on helping clients achieve their health-related goals through behavioral change. A growing body of research has investigated the effectiveness of health coaching on weight reduction [7,9,10]. However, little is known about its effectiveness among the Saudi population [11,12].

At the end of 2019, a nationwide primary-care-based health coaching program was established and implemented first in the Eastern Province, followed by other regions in Saudi Arabia. The program targets multiple behavior-related issues such as smoking, anxiety, unhealthy diet, and physical inactivity.

In the present paper, we aimed to study the effectiveness of health coaching for weight loss among the Saudi population by utilizing available data from the national health coach program in the Eastern Health Cluster (EHC).

Objectives

Our primary objective was to measure weight change before and 12 to 26 weeks after health coaching. Our secondary objectives included evaluating differences in weight change according to the type of visit (i.e., virtual, physical, and hybrid) and predictors of -5% weight loss.

## Materials and methods

Study design

This is a retrospective cohort study. The deidentified data for registered clients between 2021 and 2022 were extracted from Research Electronic Data Capture (REDCap), an online data management software hosted at King Fahad Specialist Hospital [13,14]. The study was approved by the institutional review board (IRB) at King Fahad Specialist Hospital, Dammam, Saudi Arabia (IRB study number: CLU0009). The requirement of informed consent was waived, as this is a secondary analysis of anonymized data. This report followed the STROBE cohort reporting guidelines [15].

Setting

Under the EHC umbrella, health coach clinics are distributed in 37 primary healthcare centers over a wide geographical area, including four major cities (Dammam, Qatif, Khobar, Jubail) and other cities (Safwa, Ras Tanoura, Abqaiq, Oraiarah, and Qarya Olya) denoted as rural. The clinics are accessible to all citizens, and the clients can be registered in the clinic through self-referral or healthcare provider referral. The health coaching sessions can be either in-person or virtual. These sessions are based mainly on the five A-s approach: Ask, Assess, Advise, Assist, and Arrange [16], and the health coaching process involves assessing the needs, setting the objectives, monitoring and evaluation, and providing feedback. For example, during the initial session, the health coach evaluates the client's health needs, identifies stages of change, readiness to change, and sets a Specific, Measurable, Achievable, Relevant, and Time-Bound (SMART) goal and action plan with the client. In the follow-up visits, the coach measures the success towards goal achievement and decides with the client whether to continue or change the goal. The program length and session frequency can vary according to individual needs.

Health coaches with an accredited Bachelor's degree or a two-year post-high school diploma in a health-related profession, such as nursing, health education, and nutrition, with at least one year of clinical experience, were enrolled in a four-month training program consisting of a three-month theoretical training followed by a one-month practical training, after which they were required to pass a post-training exam to be certified coaches. Training includes the following aspects: coaching principles and skills, behavior-changing theories and techniques, evidence-based practice, nutrition, physical activity, smoking cessation, mental wellness, sleep wellness, and disease management (obesity, hypertension, diabetes, dyslipidemia, osteoarthritis, and asthma).

Eligibility criteria

Any registered client aged 15 years or older, of both sexes, who had a body mass index (BMI) of 25 kg/m^2^ or higher, visited for a weight-related goal (exercise, nutritional, or weight management), and had completed at least 12 weeks of follow-up with a recorded weight between 12 to 26 weeks were eligible for inclusion. Those with unspecified visit goals, missing initial measurements of weight or height, or unknown follow-up duration were excluded.

Variables and data sources

The following variables were considered for analyses; demographic characteristics, including age, gender, and network, which is considered an indicator for residence. Data of comorbidities (type 2 diabetes, hypertension, prediabetes, prehypertension, and dyslipidemia) and smoking status (smoker or non-smoker) were also collected. The reason for health coach visits as determined by the referring care provider, the health coach, or as indicated by the client can include one or more of the following: weight management issue, nutrition issue, or physical activity issue. Moreover, the type of follow-up visits was defined as physical or virtual if all follow-up visits were of either type accordingly, or hybrid if follow-up visits were of both types. Additionally, follow-up duration is calculated as the duration in days or weeks between the initial visit and the last recorded weight-related visit, with a minimum of 78 days up to 180 days. The number of visits calculated as the total number of recorded visits was also considered for analyses. Measurements, including height and weight, were either measured by a nurse in the primary healthcare center (PHC) or reported by the client for physical and virtual visits, respectively. The BMI was calculated as weight (kg)/ height^2^ (m). Initial weight was classified into overweight for a BMI of 25 to 29.9, obesity class I for a BMI of 30 to 34.9, obesity class II for a BMI of 35 to 39.9, and obesity class III for a BMI of 40 or higher.

Primary outcomes included weight change and weight change percent. Weight change was calculated as the last recorded weight subtracted from the initial weight, while weight change percent was calculated as follows: (weight change/ initial weight) x 100.

Statistical methods

Descriptive analysis for baseline characteristics was reported as means and standard deviation (SD), or median with interquartile range (IQR) presented as 25th and 75th percentiles for continuous variables, in addition to counts and proportions for categorical variables. The paired sample t-test was performed for comparison between the initial and last recorded weight measurements. One sample t-test was used to compare the mean weight change percentage with the benchmark of -5%. Furthermore, analysis of covariance (ANCOVA) was used to compare weight change among different types of follow-ups, adjusting for the number of visits using the Bonferroni correction method. To investigate factors predicting -5% or more weight loss, logistic regression with a backward elimination method was carried out. The following variables were included in the regression: age, gender, initial weight, having weight management issue (yes, no), having at least one comorbidity (yes, no), follow-up visit type, follow-up duration in weeks, and number of visits (categorized into less than three visits, three to four visits, or five or more visits). For more robust estimates, bootstrapping was carried out for logistic regression and ANCOVA with 1000 samples. To account for missing data on the last visit weight in the predetermined duration of follow-up (78 to 180 days), the last recorded weight during this time interval was considered. The number of visits and duration of follow-up were also modified according to that visit. Moreover, outliers in the outcome variables (weight change and weight change percentage) were detected and checked. If the weight change was unrealistic based on clinical judgment and no error could be identified, the outlier was removed. The significance level was set at 0.05. Data were analyzed using SPSS version 29 (IBM Inc., Armonk, New York).

## Results

Participants and descriptive data

In total, 11,347 records were retrieved, among which 465 were included in the analysis after applying the eligibility criteria. Most participants were females (66.2%) and aged 40 years on average. Approximately one-third of the participants were classified as having obesity class I, with a median initial weight of 90 Kg (IQR: 77, 101) and a median BMI of 33.75 kg/m^2^ (IQR: 30.03, 38.69). Moreover, half of the participants reported having at least one comorbidity, including diabetes or prediabetes, hypertension or prehypertension, and/or dyslipidemia, with prehypertension being the least frequent condition (7.3%). Additionally, the majority had in-person follow-up sessions (39.8%), with a median total number of visits of three (IQR: 2,5) and a median follow-up duration of 127 days (IQR: 101, 157) (Table 1).

**Table 1:** Descriptive analysis (N=465) SD - standard deviation; IQR - interquartile range; BMI - body mass index

Variable	n (%)
Age (years), mean (SD)	40.44 (12.85)
Gender	
Male	155 (33.3%)
Female	308 (66.2%)
Network	
Dammam	158 (34.0%)
Qatif	193 (41.5%)
Khobar	27 (5.8%)
Jubail	29 (6.2%)
Rural	58 (12.5%)
Reason for health coach visit (not mutually exclusive)	
Physical activity issue	274 (58.9%)
Nutrition issue	374 (80.4%)
Weight management issue	419 (90.1)
Initial weight (kg), median (IQR)	90.00 (77, 101)
Height (cm), mean (SD)	161.65 (8.91)
Initial BMI (kg/m^2^), median (IQR)	33.75 (30.03, 38.69)
Weight classification	
Overweight	115 (24.7%)
Obesity class I	148 (31.8%)
Obesity class II	109 (23.4%)
Obesity class III	93 (20.0%)
Associated conditions (not mutually exclusive)	
Diabetes type II	93 (20.0%)
Hypertension	87 (18.7%)
Prediabetes	86 (18.5%)
Prehypertension	34 (7.3%)
Dyslipidemia	97 (20.9%)
Presence of at least 1 comorbidity	256 (55.1%)
Current smoker	34 (7.3%)
Type of follow-up visits	
Physical	185 (39.8%)
Virtual	133 (28.6%)
Hybrid	146 (31.4%)
Follow-up duration (days), median (IQR)	127.00 (101, 157)
Total number of visits, median (IQR)	3 (2,5)
Achieved at least 5% weight loss,	125 (26.9%)

Main results

Weight Change

**Figure 1:**
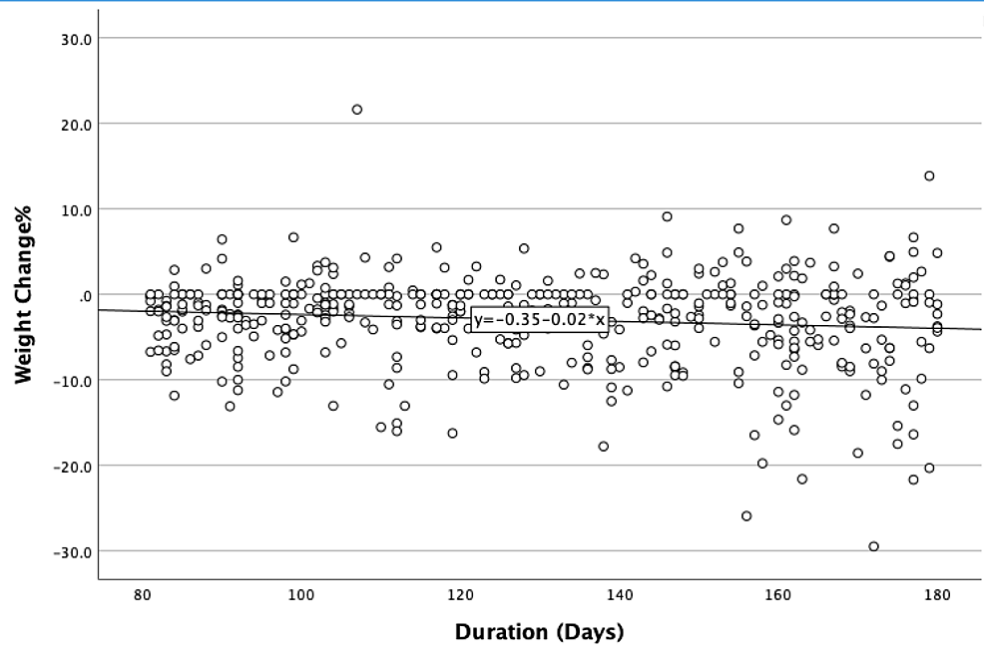
Weight change % over time

Those who completed 12 weeks or more showed a statistically significant mean difference between the initial and last weight, t (464) = -2.68 Kg (95% confidence interval [CI]: -3.12, -2.24), p<0.001, r = 0.49. The mean change percent was -2.941%, significantly different from the benchmark of -5%, t (464) = 2.06 (95% CI: 1.58, 2.54), p<0.001, r = 0.36. Moreover, 125 (26.9%) clients achieved at least -5% weight loss. The correlation between weight change percent and duration of follow-up in days is shown in Figure 1.

Weight Change Among Different Types of Follow-Up Visits

**Figure 2:**
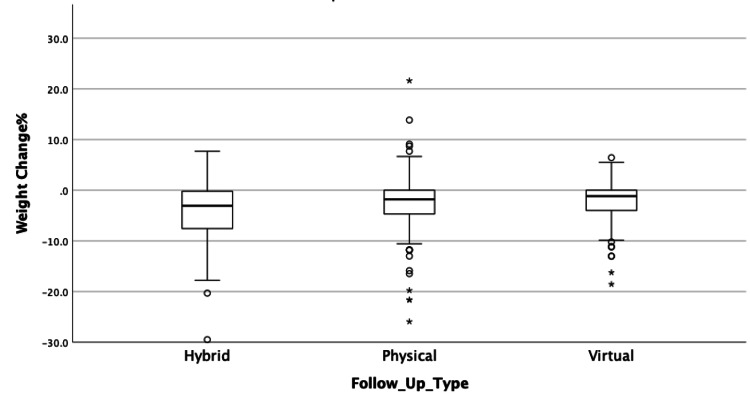
Boxplot for weight change % among types of follow-up visits

The change percent among different types of follow-ups are shown in Figure 2.

Overall, hybrid follow-up showed a greater mean weight change percent at -4.34% (SD: 5.62), physical follow-up had a mean difference of -2.34% (SD: 5.66), while virtual follow-up had -2.25% (SD: 3.99) mean weight change percent. When controlling for the total number of visits, clients who underwent physical follow-up lost an average of -2.59%, approximately similar to that of clients who underwent virtual follow-up, who lost an average of -2.54%. Those who underwent hybrid follow-up lost more, with a mean of -3.77%. Comparing each group, no statistically significant difference was found (p=0.059). Additionally, bootstrapped estimates did not show any significant difference (Table 2).

**Table 2:** Difference in weight change% between types of follow-up visits

Visit type	Mean difference	95% confidence interval		Bootstrapped 95% confidence interval	
		Lower	Upper	Lower	Upper
Physical-virtual	-0.05	-1.88	1.13	-1.12	1.09
Physical- hybrid	1.19	-0.42	2.59	-0.09	2.43
Virtual-hybrid	1.24	-0.16	3.07	-0.07	2.53

Factors Influencing -5% Weight Loss

The logistic regression model was statistically significant (X2 (5, N=453) = 65.32, p<0.001). Our model accuracy was 75.1%; however; it only explains 19.5% (Nagelkerke R squared) of the variance in the outcome (losing at least -5% of the initial weight). After adjusting for other variables in the model, the odds of losing at least -5% kg of the initial weight among males was 1.87 times higher than that among females (95% CI: 1.16, 3.02). Those who came with a weight management issue were 4.5 times more likely to lose -5% compared with those who did not (95% CI: 1.63, 12.45). Moreover, those who had at least five visits were 5.23 times more likely to lose -5% of their initial weight than those who had no more than two visits (95% CI: 2.88, 9.50). Furthermore, each additional week was associated with a 1.09 times increase in the likelihood of losing -5% of the initial weight (95% CI: 1.03, 1.15). Bootstrapping showed similar results but produced a wider 95% CI for weight management issues. Age, initial weight, having one or more comorbidity, and follow-up visit type were not significant contributing factors (Table 3).

**Table 3:** -5% Weight loss predictors (Logistic regression) aOR - adjusted odds ratio; Nagelkerke R2=0.195; (N=453, p<0.001); Constant B = -5.06

	aOR (95% confidence interval)	p-value	Bootstrapped aOR (95% confidence interval)	Bootstrapped p-value
Constant	0.006	<0.001	0.007	<0.001
Gender				
Female	Reference			
Male	1.87 (1.16, 3.02)	0.01	1.92 (1.17, 3.13)	0.006
Having weight management issue				
No	Reference			
Yes	4.50 (1.63, 12.45)	0.004	4.53 (1.90, 21.12)	0.002
Follow-up duration (weeks)	1.09 (1.03, 1.15)	0.001	1.08 (1.03, 1.15)	0.004
Total visits category				
1 to 2 visits	Reference			
3 to 4 visits	1.75 (0.95, 3.21)	0.07	1.67 (0.95, 3.25)	0.079
5 or more	5.23 (2.88, 9.50)	<0.001	5.10 (2.94, 10.07)	<0.001

## Discussion

Key results and interpretation

In this retrospective cohort study, we measured weight change in people with overweight and obesity who attended health coach clinics in primary healthcare settings at the Eastern Health Cluster, Saudi Arabia. We found a statistically significant mean difference between the initial and last weights. Weight change percent was found to be approximately -3% kg, with more than a quarter of the participants achieving at least -5% lower weight after 12 to 26 weeks of health coaching. In the literature, there are varying results with regard to achieving a clinically significant weight change after coaching. This discrepancy may be due to different coaching approaches, settings, visit frequency, and intervention duration. For example, in a retrospective study including 683 participants, the weight change after six months of online coaching was similar to that in our finding at -2.8% and -3.2% for overweight and obese individuals, respectively. At 12 months, this weight change had increased to -7.2% and -7.6%, respectively [17]. It should be noted that the median follow-up duration in our analysis was 127 days (or 18 weeks), which might not be sufficient to produce clinically significant weight loss. A randomized controlled trial (RCT) reported greater weight change exceeding -5% after a 12-week intervention [18]. This result could be attributed to RCTs settings being more strict than real-world settings.

In Saudi Arabia, a few studies have investigated lifestyle interventions for weight loss [11,12,19]. For example, in an earlier RCT with 140 participants, Alghamdi reported a mean weight loss of -5.37% after a 12-week intensive lifestyle intervention, compared with -2.62% among the education-only group [20]. Mushcab et al. piloted a pre-post lifestyle intervention adapting the CDC diabetes prevention program (DPP) over two phases. They found a mean difference of -3.3 kg and -5.1 kg at six and nine months, respectively [21]. The difference between these studies could be linked to the specific diet and exercise routine used in each study [20,21].

We further compared the weight change among different types of follow-ups, which was statistically insignificant. Several studies showed comparable findings [22-25]. For instance, a study that involved coaching based on motivational interviewing with a sample size of 92 participants found no difference in weight change between the virtual and in-person coaching groups after eight weeks [22]. Other studies that incorporated the DPP curriculum and compared the telehealth and face-to-face delivery methods also reported no difference in weight change between both groups [23,24]. On the other hand, Johnson et al. reported that online coaching was significantly more effective, and they attributed the result to the lower attendance rate in the in-person group [25]. The reported results in these studies, along with our results, support that tele-coaching is not inferior to on-site coaching. However, our finding should be interpreted cautiously due to the possibility that we excluded more clients in the virtual group than in the physical group, as they are more likely to have less than two weight measurements. The hybrid group achieved higher weight loss, but this difference was deemed insignificant after controlling for total visits. Nonetheless, the confidence intervals of the post hoc analysis for comparisons between the hybrid and other visit types were wide, suggesting the possibility that hybrid visits might be significantly better with a larger sample size.

Several studies have investigated weight change predictors in lifestyle interventions [26,27]. The present study explored the factors predicting weight loss of at least -5% kg and showed that gender, total visits, follow-up duration, and having a weight management goal, among other factors, contributed to the possibility of -5% kg weight loss. Being male was associated with achieving greater weight loss, a finding reported by many studies [26-28]. In addition to the supportive findings from the literature, this finding could also be attributed to the lower physical activity among Saudi women [29,30]. In our analysis, the length of follow-up was positively associated with successful weight loss. However, further investigation is needed to examine this finding over a longer duration. A systematic review that included lifestyle intervention studies with at least one year of the intervention found that weight loss decreased over time [31]. The total number of visits was the greatest influencing factor for -5% weight loss, particularly a total number of coaching visits of more than four. Higher session attendance rate and visit frequency are well-documented predictive factors for weight loss in a number of studies [32-35]. Noteworthy, in the present study, the median number of visits was only three sessions, suggesting that more follow-up sessions might be needed in this national program to increase the success rates of weight loss. Moreover, having a weight management issue as a reason for the initial coaching visit was the second most important predictive factor. In this health coaching program, some clients might visit only for diet or exercise but not for weight loss; thus, they do not focus on weight management. In addition, several other weight loss-influencing factors have been reported. For example, early weight loss was reported by many studies to be a significant and strong contributing factor for greater weight change [26,27,36-38]. Additionally, self-motivation, regular weight monitoring, and weekly food logs were found to be associated with weight loss [26,33].

Limitations

Our study has several limitations. First, owing to the nature of the retrospective design, we could not control for some factors that could influence weight loss. For example, data about receiving other interventions were not available. In addition, this study was based on secondary data, not primarily collected data for research purposes; hence, data quality could be an issue. The lack of a control group presents another limitation. Moreover, because the analysis was limited to those who had weight measurements between 12 to 26 weeks, there is a high possibility of selection bias, specifically; attrition bias. Indeed, many studies on lifestyle interventions have reported a high loss to follow-up rate [39]. Additionally, the participants were limited to one region in Saudi Arabia, and the duration of follow-up might not be sufficient for a clinically significant outcome to occur.

Generalizability

Being a multi-center study with a large sample size in real-world settings might support the generalizability of our results. However, because the analysis was limited to those with weight measurements between 12 and 26 weeks, our results might not be generalizable to those who dropped out before the 12th week. Additionally, the study was limited to one region in Saudi Arabia and may not be generalizable to other regions.

## Conclusions

We found a statistically significant weight change among clients who completed 12 weeks of coaching in primary care settings. The findings in this paper contribute to the importance of lifestyle interventions for weight loss among the Saudi population. However, stronger prospective and controlled studies with longer follow-ups are needed to confirm this finding.
